# Nrf2 Activation Does Not Protect from Aldosterone-Induced Kidney Damage in Mice

**DOI:** 10.3390/antiox12030777

**Published:** 2023-03-22

**Authors:** Ronja Brinks, Christoph Jan Wruck, Jutta Schmitz, Nicole Schupp

**Affiliations:** 1 Institute of Toxicology, Medical Faculty, University of Düsseldorf, 40225 Düsseldorf, Germany; 2 Department of Anatomy and Cell Biology, Uniklinik RWTH Aachen, 52074 Aachen, Germany

**Keywords:** aldosterone, hypertension, kidney injury, oxidative stress, Keap1 knockdown

## Abstract

Nuclear factor erythroid 2-related factor 2 (Nrf2) is downregulated in chronic kidney disease (CKD). Activation of Nrf2 might be a therapeutic option in CKD. Here we investigate the effect of Nrf2 activation on aldosterone (Aldo)-induced renal injury. Wild-type (WT) mice, transgenic Keap1 hypomorphic (Nrf2ꜛ, genotype results in upregulation of Nrf2 expression) mice and WT mice treated with the Nrf2 activator sulforaphane (Sulf) received Aldo for 4 weeks. In Aldo-treated mice, kidneys were significantly heavier and pathologically altered, reflected by increased urinary albumin levels and tissue damage. In Nrf2ꜛ-Aldo mice the tubule damage marker NGAL was significantly decreased. Increased oxidative damage markers (8-OHdG, 15-isoprostane F_2t_) were measured in all Aldo-treated groups. Aldo-increased Nrf2 amounts were mainly found in the late tubule system. The amount of phosphorylated and thus putatively active Nrf2 was significantly increased by Aldo only in WT mice. However, expression of Nrf2 target genes NQO1 and HO1 was decreased in all Aldo-infused mice. GSK3β, which promotes Nrf2 degradation, was significantly increased in the kidneys of Aldo-treated WT mice. Neither genetic nor pharmacological Nrf2 activation was able to prevent oxidative injury induced by Aldo, probably due to induction of negative regulators of Nrf2.

## 1. Introduction

The main risk factors for the development of chronic kidney disease (CKD) are diabetes and hypertension. Hypertension is present in approximately 70% of individuals with moderate CKD in Germany [1]. In hypertension, the renin–angiotensin–aldosterone system (RAAS) is almost always activated, and is usually also causally involved in the development of hypertension. Therefore, treatment with RAAS inhibitors such as angiotensin converting enzyme inhibitors or mineralocorticoid receptor blockers is standard therapy for achieving the first therapeutic goal in hypertensive CKD patients, the lowering of systolic blood pressure to 120 mmHg [2]. Beyond that, the therapeutic options for treating CKD and especially stopping the progression of CKD are limited.

Several clinical studies focused on investigating the effects of activators of the transcription factor nuclear factor erythroid 2-related factor 2 (Nrf2) on progression of mainly diabetes-induced CKD [3,4]. This is based, on the one hand, on results of animal studies in which Nrf2 activation led not only to improved renal morphology, but also to an increase in renal function [5]. On the other hand, an increased glomerular filtration rate was observed in clinical antitumor studies after intake of the Nrf2 activator bardoxolone methyl (CDDO-Me) [6]. However, initial larger studies in CKD patients showed serious cardiac side effects of CDDO-Me, increased proteinuria and no arrest of CKD progression [3,7]. Recent studies include only patients without risk factors for heart failure [8]. It was suggested that the actual effects of Nrf2 activators in the kidney are not yet sufficiently understood for use of these compounds in the clinic [9,10].

In rats with aldosterone (Aldo)-induced hypertension and moderate kidney damage we found that the use of another Nrf2 activator, sulforaphane (Sulf), protected kidneys from injury and oxidative damage [11]. As an isothiocyanate, Sulf belongs to another substance class than CDDO-Me, which is a synthetic triterpenoid, and as a natural compound Sulf is found in cruciferous vegetables and was isolated from broccoli [12]. CDDO-Me and Sulf, being electrophilic compounds, activate Nrf2 by inhibiting its repressor Kelch-like ECH-associated protein 1 (Keap1) through interaction with one of its cysteine groups, as do all other Nrf2 activators currently in clinical trials [13]. Due to their reactivity towards thiols, these substances are not specific Nrf2 activators but can affect many targets and signaling pathways [14]. New, specific protein–protein inhibitors of the Keap1–Nrf2 interaction are being explored at the moment but have not yet reached the clinic [13]. We have recently shown that in mice with moderate kidney injury caused by increased Aldo concentrations the expression of Nrf2 target genes in kidney cells was significantly reduced despite an increased abundance of Nrf2 and its putatively activated phosphorylated form in the kidney [15]. Similar findings have been observed in patients with advanced CKD [16] and also in animal models of CKD [5,17].

Nrf2 is ubiquitously expressed in tissues and cells and, upon activation, induces transcription of antioxidant enzymes and enzymes of metabolism in response to oxidative stress or appearance of electrophiles [18]. However, Nrf2 plays a role not only in induced stress but also in maintaining redox homeostasis under physiological conditions [19]. Nrf2 belongs to the basic leucine zipper (bZIP) transcription factor family and is regulated transcriptionally, posttranscriptionally and posttranslationally [20]. Under normal physiological conditions, Nrf2 is localized in the cytoplasm and bound to its repressor Keap1 [18]. Binding of Nrf2 by Keap1 induces its degradation via the ubiquitin ligase complex (Cul3/Rbx1) [21]. However, if the cell is under oxidative stress or electrophiles are present, the thiol groups of the cysteine residues of Keap1 are modified, resulting in a conformational change in Keap1, preventing ubiquitin transfer. Keap1 inactivation thus leads to stabilization, accumulation and translocation of Nrf2 into the nucleus [22]. Furthermore, phosphorylation of Nrf2 via diverse kinases can also affect binding to Keap1. For example, it has been shown that phosphorylation of Nrf2 at serine 40 via oxidative stress-activated PKC leads to stabilization of Nrf2 and subsequent translocation to the nucleus [23]. In the nucleus, Nrf2 forms a heterodimer with small Maf (sMaf) proteins (MafF, MafG and MafK). Formation of the dimer is necessary for binding to the antioxidant responsive element (ARE) and induction of transcription of Nrf2 target genes [24]. However, there are other Keap1-independent mechanisms that may influence Nrf2 activity. For example, the kinase Fyn, which is activated via the kinase glycogen synthase kinase 3β (GSK3β), has been shown to phosphorylate Nrf2 in the nucleus, which in turn leads to export of Nrf2 out of the nucleus and its degradation [25]. GSK3β can also phosphorylate Nrf2 directly, leading to stabilization but also degradation of Nrf2 [26]. After phosphorylation by GSK3β, ubiquitination occurs via β-transducin repeat-containing protein (β-TrCP) followed by degradation in the proteasome [25]. Furthermore, the Nrf2 heterodimer in the nucleus competes for binding at the ARE with sMaf hetero- or homodimers and the Bach1/sMaf heterodimer. In this context, binding of the BTB and CNC homology 1, basic leucine zipper transcription factor 1 (Bach1)/sMaf dimer acts to repress transcription of Nrf2-regulated genes.

Therefore, in the present work, we investigate the effects of both pharmacological (using Sulf) and genetic Nrf2 activation on moderate renal injury triggered by elevated levels of Aldo with a focus on the difference in the two approaches on kidney parameters and the Keap1-independent regulation of Nrf2. Sulf was chosen as the Nrf2 activator in this study because, although it has the same mode of action as CDDO-Me mentioned above, it belongs to a different class of compounds, has shown promising effects in the trial with our rats and has to date shown no severe adverse effects in the more than 70 clinical trials in which it has already been used [14].

## 2. Materials and Methods

### 2.1. Animal Treatment

Thirty-six male C57BL/6-mice (Janvier, LE Genest Saint Isle, France) were allocated randomly to four equal-sized groups at the age of 12 weeks. Additionally, 16 B6.Cg-Keap1^tm2Mym^(Alb-cre)21Mgn/J mice (from here on named Nrf2ꜛ), negative for Alb-cre (genotyped with the primers in Table S1), were divided into two age-matched groups of 8 animals each at an average age of 12 weeks. The strain was generated by crossing Keap1^loxP/loxP^ with mice expressing Cre recombinase under the control of the albumin (Alb) promoter by Okawa et al. [27]. Osmotic minipumps were implanted (Model 1004, Alzet, Durect Coporation, Cupertino, CA, USA) subcutaneously in the neck region of the mice under inhalant isoflurane anesthesia (1.5–2%, anesthesia station MiniTAG, TEM SEGA, Pessac, France). The minipumps administered 125 µg Aldo/kg × day for 28 days. The control group received 15% EtOH in PBS as a solvent control. In addition, all mice had free access to food and 1% (*w*/*v*) NaCl as drinking water. Eight of the WT mice infused with Aldo or solvent also received an average of 10–20 mg/kg sulforaphane (Sulf) per day, dissolved in 1% NaCl, for 28 days, depending on their drinking volume. Preemptive analgesia was achieved with 5 mg/kg carprofen (Zoetis Deutschland GmbH, Berlin, Germany). The blood pressure was measured non-invasively twice weekly using the tail cuff method (Visitech Systems, Apex, NC, USA). The mice were habituated to the blood pressure measurement procedure 2 weeks before implantation of minipumps. At the beginning and the end of the experiment, the mice were placed into metabolic cages for 20 h to collect urine samples. After 4 weeks of treatment, mice were deeply anesthetized (120 mg ketamine/kg and 8 mg xylazine/kg i.m.) and perfused with ice-cold Deltadex 40 (AlleMan Pharma GmbH, Rimbach, Germany) supplemented with 1% procaine hydrochloride (bela-pharm, Vechta, Germany), followed by ice-cold 0.9% NaCl solution (Fresenius Kabi Deutschland GmbH, Bad Homburg, Germany). Kidneys and hearts were removed, weighed and either embedded in paraffin or snap-frozen in liquid nitrogen and stored at −80 °C.

### 2.2. Quantification of Aldosterone

Serum aldosterone levels were measured using the Aldosterone ELISA Kit (BT E-5200, BioTrend, Cologne, Germany) as instructed by the manufacturer.

### 2.3. Parameters of Renal Function

Renal function was assessed by measuring serum creatinine, calculating creatinine clearance and quantifying excretion of albumin, kidney injury molecule-1 (KIM-1) and neutrophil gelatinase-associated lipocalin (NGAL). Creatinine in urine and serum was determined using creatinine urinary/serum colorimetric assay kits (No. 500701/700460, Cayman Chemical Company, Ann Arbor, MI, USA) according to the manufacturer’s protocol. The mouse albumin ELISA kit (EMA3201-1, Assay Pro, St. Charles, IL, USA), the mouse KIM1/TIM1 ELISA kit (PicoKineTM, EK0880, BosterBio, Pleasanton, CA, USA) and the mouse NGAL ELISA kit (KIT 042, BioPorto, Gentofte, Denmark) were used for the quantification of albumin, KIM-1 and NGAL excretion following the manufacturer’s protocol. Albumin, KIM-1 and NGAL were related to urinary creatinine.

### 2.4. Histopathology

For histopathological examinations of the kidney, 3 µm paraffin sections were stained with hematoxylin and eosin, periodic acid–Schiff stain and Sirius red, which was also performed on heart sections. Both tubular and glomerular damage were determined as previously described [28].

### 2.5. Immunohistochemistry

Kidney sections (3 µm) (RM 2164, Leica, Wetzlar, Germany) were mounted on glass slides, heated at 60 °C for 1 h and deparaffinized with Roti-Histol (Roth, Karlsruhe, Germany) and ethanol. Antigen retrieval was performed with citrate buffer (DAKO Retrieval Solution, pH 6.0, Agilent Technologies, Santa Clara, CA, USA) at 95 °C for 30 min. Slides were then blocked and incubated overnight at 4 °C with the appropriate primary antibodies. The specific antibodies and dilutions were as follows: anti-γ-H2AX (#9718, 1:200, Cell Signaling, Herts, UK), anti-Nrf2 (sc-722, 1:1000, Santa Cruz Biotechnology, Dallas, TX, USA) and anti-pNrf2 (S40, ab76026, 1:1000, abcam, Cambridge, UK). Sections were next incubated with the biotinylated secondary goat anti-rabbit antibody (ab6720, 1:200, abcam, Cambridge, UK) for 45 min at room temperature. Antibody binding was visualized as previously described [11,29]. Sections were counterstained with hematoxylin. Images were acquired at 200-fold magnification. The ratio of positive to negative nuclei or areas was scored via ImageJ [30] within 10 visual fields of the cortex and 3–5 visual fields of the medulla.

### 2.6. Double Staining

To localize pNrf2 in the kidney, double staining was performed to identify pNrf2 positives in different types of kidney cells. Staining of the first antigen (pNrf2) was performed as described above using diaminobenzidine (DAB) as a chromogen. After visualization of antibody binding, the protocol was repeated with an antibody against calbindin (1:200, #2173, Cell Signaling, Herts, UK) to identify distal tubular cells and cells of the early collecting duct. Proximal tubular cells were identified by the presence of the brush border, whereas glomeruli were identified by their capillary tuft (blue circles). Fifty glomeruli were analyzed per animal. The late collecting duct was identified by the absence of positive calbindin staining and brush border. The VECTOR^®^ VIP Peroxidase Substrate Kit was used to visualize the second antigen (SK-4600, Vector Lab, Burlingame, CA, USA), which produced a purple stain. Sections were counterstained with hematoxylin and dehydrated in descending alcohol concentrations. Pictures were taken at 400-fold magnification. The ratio of positive to negative nuclei for pNrf2 on 10 visual fields was assessed via ImageJ [30] by counting only nuclei positive for the specific kidney cell identifier.

### 2.7. Quantification of 8-OhdG and 15-Isoprostane F_2t_ in Urine

8-hydroxy-2′-deoxyguanosine (8-OHdG) in urine was quantified using the DNA Damage ELISA Kit (StessMarq Biosciences Inc., Victoria, BC, Canada) according to the manufacturer’s protocol. Urinary 15-isoprostane F_2t_ levels were determined with the Urinary Isoprostane ELISA Kit (EA85, Oxford Biomedical Research, Rochester Hills, MI, USA) following the manufacturer’s protocol. 15-isoprostane F_2t_ was related to urinary creatinine.

### 2.8. Western Blot

For protein isolation, frozen kidney tissue was manually pestled and lysed in RIPA buffer (50 mM Tris, 150 mM NaCl, 1 mM EDTA, 0.025% Natriumdesoxycholat, 1% Nonidet, 1 mM NaF) supplemented with a protease inhibitor cocktail (Sigma, Taufkirchen, Germany) and a phosphatase inhibitor cocktail (Thermo Scientific, Rockford, IL, USA). After a centrifugation step at 10,000× *g* for 15 min at 4 °C, protein extracts were stored at −80 °C. Subsequently, 50 µg protein was loaded onto an SDS gel and transferred to a nitrocellulose membrane (GE Healthcare, Little Chalfont, UK) after separation. Membranes were incubated with specific primary antibodies against Bach1 (abx322188, Abbexa, Cambridge, UK), FYN (ab184276, abcam, Cambridge, UK), γGCLC (PA1492, BosterBio, Pleasanton, CA, USA), GSK3β (#9315, Cell Signaling, Herts, UK), pGSK3β (#9336, Cell Signaling, Herts, UK), HO1 (ab13243, abcam, Cambridge, UK), Keap1 (ab227828, abcam, Cambridge, UK), MafK (GTX129240, GeneTex, Irvine, CA, USA), NQO1 (ab34173, abcam, Cambridge, UK), Nrf2 (sc-722, Santa Cruz Biotechnology, Dallas, TX, USA), pNrf2 (ab76026, abcam, Cambridge, UK), SOD1 (GTX100554, GeneTex, Irvine, CA, USA), TrxR1 (GTX108727, GeneTex, Irvine, CA, USA) and, as housekeepers, α-tubulin (sc-5286, Santa Cruz Biotechnology, Dallas, TX, USA), GAPDH (#2118, Cell Signaling, Herts, UK) and lamin B2 (#2328, Cell Signaling, Herts, UK) overnight at 4 °C, followed by an incubation with HRP-conjugated secondary antibodies for 2.5 h at room temperature. Antibody binding was visualized using the BM Chemiluminescence Blotting Substrate Kit (Roche, Basel, Switzerland) according to the manufacturer’s instructions. Chemiluminescence signals were recorded using the ChemiDoc™ Touch Imaging System (BIO-RAD, Hercules, CA, USA).

### 2.9. Preparation of Cytosolic and Nuclear Protein Fractions

To detect translocation of specific proteins into the nucleus, a cytosolic and a nuclear fraction were prepared. To the cytosolic (10 mM Tris-HCl, 50 mM NaCL, 500 mM sucrose, 0.1 mM EDTA, and 0.5% Triton-X) and nuclear lysis buffers (10 mM Tris-HCl, 500 mM NaCL, 0. 2 mM EDTA, 1% Nonident P40, and 1% Tergitol) were added 1 mM DTT, 1 mM PMSF, 1 mM sodium orthovanadate and 1× protease inhibitor cocktail (Sigma, Taufkirchen, Germany) just before the start of the experiment. All work was performed at 0–4 °C. For extraction, frozen kidney tissue was first minced and homogenized with a pestle in cytosolic lysis buffer for 2 min and then incubated for an additional 5 min. The homogenate was centrifuged at 1000× *g* for 10 min to separate the nuclear proteins (pellet) from the cytosolic proteins (supernatant). After a wash step, the supernatant representing the cytosolic fraction was removed and stored on ice. After another centrifugation (1000× *g*, 4 min), the remaining pellet was resuspended in 300 µL of cytosolic lysis buffer. After washing, the pellet was resuspended in 150 µL of nuclear lysis buffer and sonicated. After centrifugation at 14,000× *g* for 10 min, the supernatant representing the nuclear fraction was removed. Fifty micrograms of the protein lysate of the cytosolic fraction and 10 µg of the protein lysate of the nuclear fraction were used for a Western blot. The cytosolic and nuclear fractions were stored at −80 °C until further use.

### 2.10. Quantitative RT-PCR

mRNA was extracted from 20–40 mg frozen kidney tissue using the QIAcube (Qiagen, Hilden, Germany) according to the manufacturer’s instructions. Isolated mRNA was transcribed into cDNA using the High Capacity cDNA Reverse Transcription Kit (Thermo Fisher, Waltham, MA, USA). Quantitative Real-Time PCR (qRT-PCR) was performed with 20 µg cDNA and the SensiMix SYBR Hi-ROX Mastermix (Bioline GmbH, Luckenwalde, Germany) using the CFX96 Real-Time System (BIO-RAD, Hercules, CA, USA). Primer sequences utilized for gene expression analysis are listed in Table S2. Relative expression levels of target genes were normalized to the housekeepers GAPDH and β-actin and calculated using the comparative CT method with the analysis software Bio-Rad CFX Manager 3.1 (BIO-RAD, Hercules, CA, USA).

### 2.11. Statistics

Data from seven to eight animals per group are expressed as mean ± standard error of the mean (SEM). GraphPad Prism 6 (GraphPad Software, La Jolla, CA, USA) was used for statistical analyses. Data were tested for normal distribution using the Kolmogorov–Smirnov test with Dallal–Wilkinson–Liliefor *p* values. Groups of each mouse strain were tested for significance (normal distribution) among themselves or against control using analysis of variance (one-way ANOVA) followed by Tukey’s correction. Values not normally distributed were tested for significance using the Kruskal–Wallis test followed by Dunn’s multiple comparison test. Significant differences between mouse strains (controls or Aldo-infused animals) were tested for significance using 2-way ANOVA followed by Tukey’s correction. A *p* value ≤ 0.05 was considered significant.

## 3. Results

### 3.1. Characteristics of Keap1 Hypomorphic (Nrf2ꜛ) Mice

**Figure 1:**
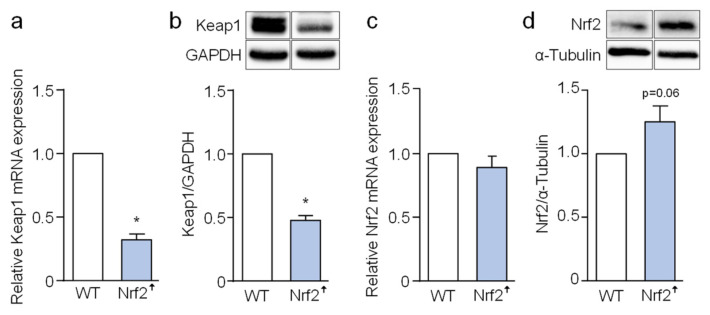
Expression of Keap1 and Nrf2 in the transgenic hypomorphic Keap1 mice (Nrf2ꜛ). mRNA expression of *Keap1* (**a**) and *Nrf2* (**c**) in kidneys evaluated via RT-PCR. Representative picture of the Western blots of the expression of Keap1 (70 kDa, (**b**)) and total Nrf2 (95–110 kDa, (**d**)) in kidneys of mice as well as the quantification of band densities of the above-mentioned protein measured via ImageJ and related to the housekeeper GAPDH (37 kDa). Data are shown as mean + SEM. *n* = 8. * *p* ≤ 0.05 vs. WT.

Hypomorphic Keap1 (Nrf2ꜛ) mice showed significantly decreased expression of Keap1 in the kidney at both the mRNA and protein levels (Figure 1a,b). This did not affect *Nrf2* gene expression (Figure 1c), which was not expected because Keap1 regulates Nrf2 primarily posttranslationally. However, an increased amount of Nrf2 protein could be measured in the mice (Figure 1d).

### 3.2. Blood Pressure Changes and Clinical Characteristics

**Figure 2:**
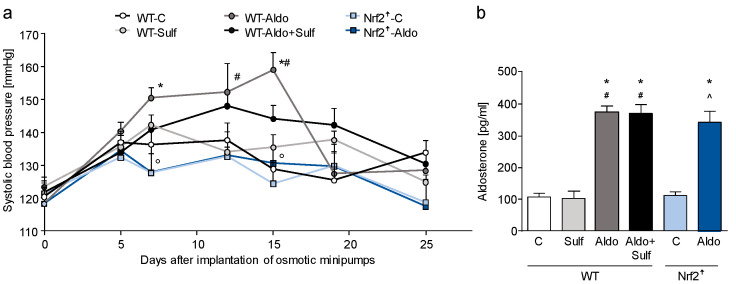
Blood pressure changes and aldosterone levels. (**a**) Development of systolic blood pressure in the different treatment groups. The blood pressure was measured at six time points after acclimatization to the measurement procedure. The first time point represents baseline blood pressure before implantation of the osmotic pumps. (**b**) Blood aldosterone levels after 28 days of treatment. Aldo: aldosterone, C: control, Nrf2: nuclear factor erythroid 2-related factor 2, Sulf: sulforaphane, WT: wild type. Data are shown as mean + SEM. *n* = 8. * *p* ≤ 0.05 vs. WT-C, ^#^ *p* ≤ 0.05 vs. WT-Sulf, ^^^ *p* ≤ 0.05 vs. Nrf2ꜛ-C.

Only the WT mice infused with 125 µg/kg Aldo per day showed a temporary significant increase in blood pressure up to 155 mmHg until the 15th day after implantation (Figure 2a). After this time point, however, the blood pressure decreased to the level of the WT control (C) group by the end of the experiment. In the Aldo-infused WT mice treated additionally with Sulf in the drinking water, blood pressure did not increase above the level of the WT control. Quantification of the aldosterone levels in the serum of the six mouse groups showed a significant increase in all Aldo-treated groups (Figure 2b).

A difference in body weight could not be detected between the six different groups (Table 1). However, compared to the WT mice, the Nrf2ꜛ mice had a higher kidney weight already basally. Aldo infusion in the WT mice and in the Nrf2ꜛ mice resulted in a significant increase in relative kidney weight. Aldo treatment in the Nrf2ꜛ mice resulted in an even greater increase in kidney weight, which was even significantly higher than in the Aldo-infused WT mice. Additional treatment of the Aldo-exposed WT mice with Sulf also resulted in an increase in kidney weight compared with the WT control and Aldo-only treatment. The relative heart weights of WT control and Nrf2ꜛ control mice did not differ basally. Sulf had no impact on relative heart weight, but Aldo infusion resulted in a significant increase in relative heart weight compared with WT control in both WT and Nrf2ꜛ mice. Collagen deposition was significantly increased in Aldo-treated Nrf2 animals, while Aldo-treated WT animals showed no change at all.

### 3.3. Histopathological Changes in the Kidney and Renal Function

During organ removal, hydronephrosis of the right kidney was observed in two of eight mice in the Nrf2ꜛ control group and in four of eight mice in the Nrf2ꜛ-Aldo-infused group (these kidneys were not used for further analyses). Significant pathologic damage to the tubular system was recorded in Aldo-treated WT animals, and included infiltration of inflammatory cells, atrophy of basement membranes and fibrotic changes (Table 1, TSI). Nrf2ꜛ mice were not affected, neither basally nor by Aldo. Aldo-treated WT mice also showed a significant increase in mesangiolysis (Table 1, MSI), whereas all mice, including those treated only with Sulf and the Nrf2ꜛ control, presented increased glomerular sclerosis (Table 1, GSI).

Aldo treatment resulted in a significant increase in drinking and urine volume (Table 1) in all groups compared with the matched controls and the control of the respective other mouse strain (WT-C vs. Nrf2ꜛ-Aldo and Nrf2ꜛ-C vs. WT-Aldo). Creatinine in serum was not different between the groups. Aldo alone did not affect creatinine clearance. However, treatment with Sulf resulted in significantly lower creatinine clearance compared with WT control and combination treatment (WT-Aldo + Sulf) and the Nrf2ꜛ mice showed a slightly lower creatinine clearance basally. Albumin was used as a marker for glomerular damage in this experiment, while urinary NGAL and KIM-1 served as markers of tubular damage. All Aldo-treated animals had significantly elevated albumin levels, with the Nrf2ꜛ-Aldo group presenting the highest levels. Aldo infusion resulted in a tenfold increase in KIM-1 to creatinine ratio in WT mice only, whereas Nrf2ꜛ mice treated with Aldo had significantly lower levels. All Aldo-treated animals had significantly elevated NGAL levels, with the Nrf2ꜛ-Aldo group here presenting the lowest levels. In the co-treated mice, Sulf treatment had no effect at all on the three markers.

### 3.4. Oxidative Stress Markers

**Figure 3:**
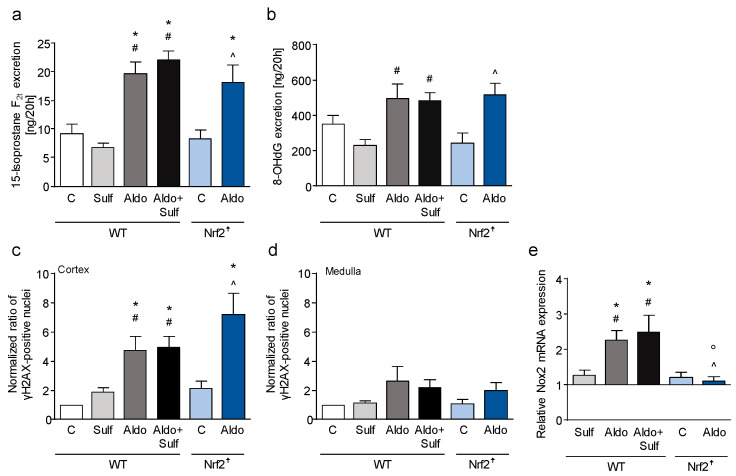
Detection of markers of oxidative damage in collected urine and kidney tissue of Aldo-infused WT and Nrf2ꜛ mice and control mice after 28 days of treatment. (**a**) Amount of 15-isoprostane F_2t_ formed as a result of lipid peroxidation. (**b**) Amount of oxidized and excised/eliminated base 8-OHdG. Quantification of DNA damage in (**c**) cortex and (**d**) medulla on paraffin-embedded kidney sections stained with an antibody against γ-H2AX, a marker of structural DNA damage. (**e**) mRNA expression of Nox2 measured by RT-PCR. 8-OHdG: 8-hydroxy-2′-deoxyguanosine, γ-H2AX: phosphorylated histone 2AX, Aldo: aldosterone, C: control, Nox2: NADPH oxidase isoform 2, Nrf2: nuclear factor erythroid 2-related factor 2, Sulf: sulforaphane, WT: wild-type. Data are shown as mean + SEM, *n* = 7–8. * *p* ≤ 0.05 vs. WT-C, ^#^ *p* ≤ 0.05 vs. WT-Sulf, ° *p* ≤ 0.05 vs. WT-Aldo, ^ *p* ≤ 0.05 vs. Nrf2ꜛ-C.

Systemic oxidative stress was determined by the excretion of 15-isoprostane F_2t_, a marker of lipid peroxidation, and 8-OHdG, a marker of oxidative damage to nucleic bases, in the collection urine of mice. 15-isoprostane F_2t_ was significantly increased in all Aldo-treated animals. Higher 8-OHdG excretion was detected in WT and Nrf2ꜛ mice compared with the WT-Sulf group and the Nrf2ꜛ-C group, respectively (Figure 3a,b). As another reasonably stable marker for oxidative stress, the DNA damage marker γH2AX was quantified on paraffin sections of the kidneys. Here, a localization of damage in renal cortex and medulla is feasible. Compared with the WT-C group, there were approximately twice as many γH2AX-positive nuclei in the renal cortex of Sulf-treated WT mice and in the Nrf2ꜛ-C group (Figure 3c,d). Aldo resulted in a significant increase of 4- and 6-fold in γH2AX-positive nuclei in all three Aldo-treated groups compared with the respective control groups. In the medulla, there was no elevated damage after Aldo treatment. As a possible source of oxidative stress, we examined the change in expression of NADPH oxidase isoform 2, which was significantly upregulated in the two Aldo-treated WT groups (Figure 3e).

### 3.5. Nrf2 Expression, Activation and Target Gene Regulation

**Figure 4:**
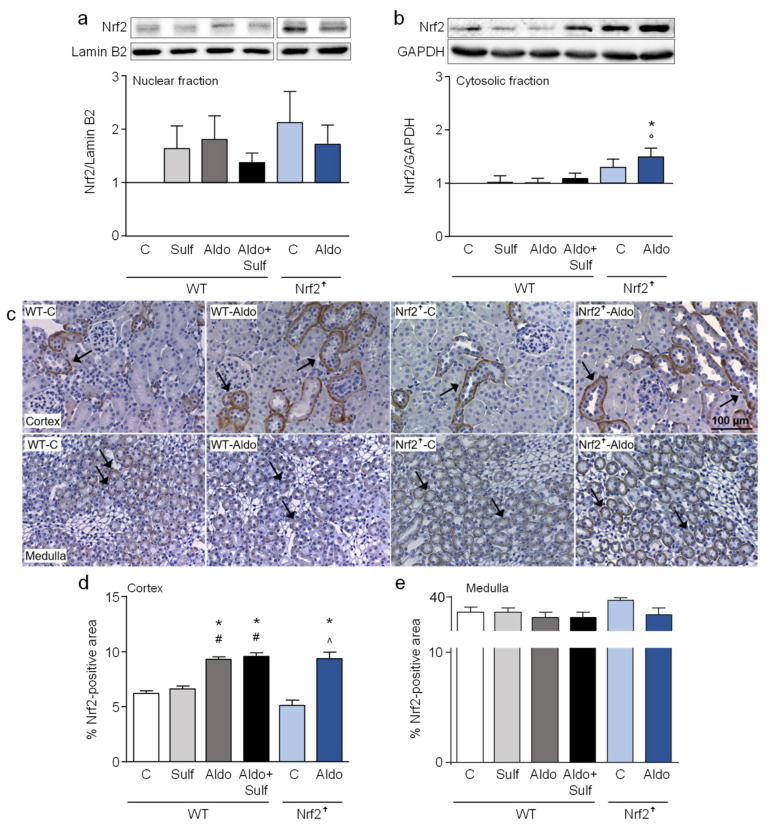
Abundance of Nrf2 in Aldo-infused WT and Nrf2ꜛ mice and control mice after 28 days of treatment. Representative pictures of the Western blots of the expression of total Nrf2 (95–110 kDa) in (**a**) nuclear and (**b**) cytosolic extracts of kidneys of mice as well as the quantification of band densities of the above-mentioned protein measured via ImageJ and related to the housekeepers lamin B2 (68 kDa) and GAPDH (37 kDa). (**c**) Representative pictures of cortical and medullary kidney sections from control and aldosterone-infused animals stained with an antibody against Nrf2. Examples of positive stained areas are marked with black arrows. Percentage of Nrf2-positive stained areas in cortex (**d**) and medulla (**e**). For the quantification of positive Nrf2 areas, 10 visual fields of cortical and 3–5 visual fields of medullary kidney sections were analyzed per animal via ImageJ. Aldo: aldosterone, GAPDH: glyceraldehyde 3-phosphate dehydrogenase, Nrf2: nuclear factor erythroid 2-related factor 2, Sulf. sulforaphane, WT: wild-type. Data are shown as mean + SEM, *n* = 7–8. * *p* ≤ 0.05 vs. WT-C, ^#^ *p* ≤ 0.05 vs. WT-Sulf, ° *p* ≤ 0.05 vs. WT-Aldo, ^ *p* ≤ 0.05 vs. Nrf2ꜛ-C.

No significant difference in Nrf2 abundance in nuclear and cytosolic kidney samples was observed between groups, except for the Aldo-treated Nrf2ꜛ group (Figure 4a,b). Here, the amount of cytosolic Nrf2 was significantly increased compared with the WT control and the WT-Aldo group. To obtain better local resolution of Nrf2 levels, renal tissue was immunohistochemically stained with an antibody against Nrf2 (Figure 4c–e). A 4- to 8-fold higher expression of Nrf2 was observed in the medulla compared with the cortex, but significantly altered abundance of Nrf2 was observed only in the cortex, increased in all Aldo-treated groups.

**Figure 5:**
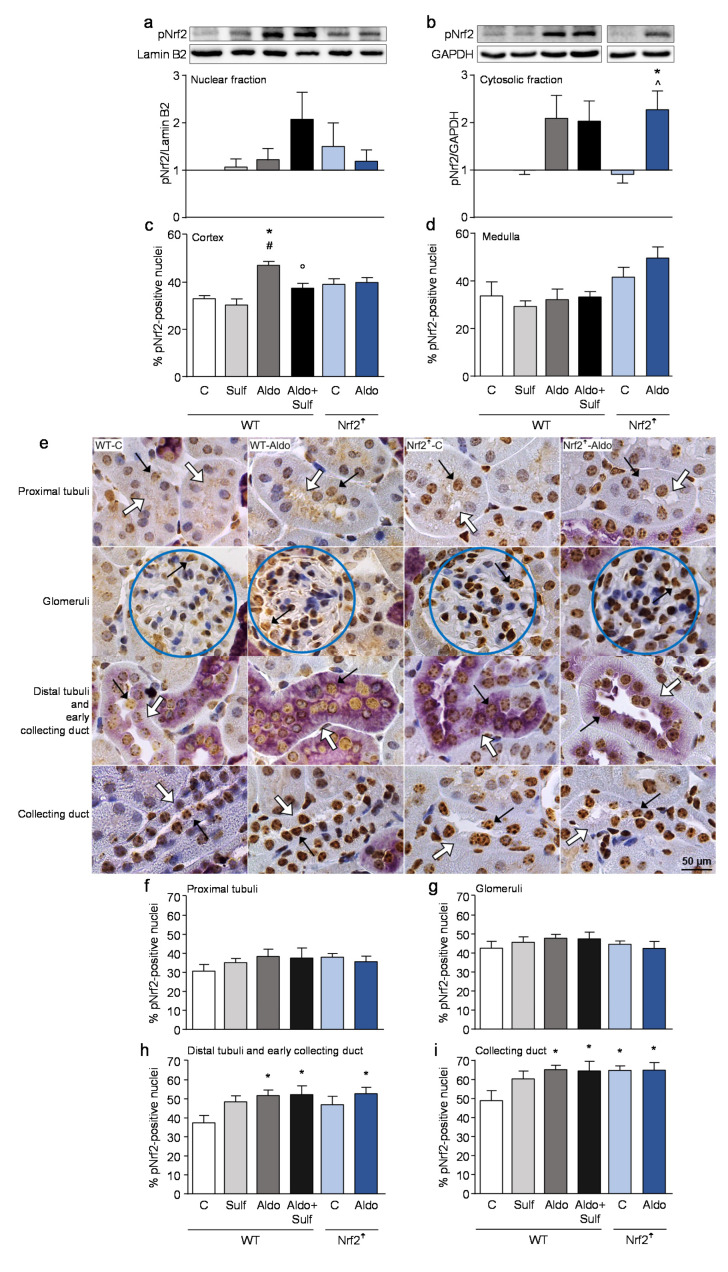
Localization of the activated transcription factor Nrf2. Representative pictures of the Western blots of the expression of total Nrf2 phosphorylated at Ser40 (pNrf2, 110 kDa) in (**a**) nuclear and (**b**) cytosolic extracts of kidneys of mice as well as the quantification of band densities of the above-mentioned protein measured via ImageJ and related to the housekeepers lamin B2 (68 kDa) and GAPDH (37 kDa). Percentage of pNrf2-positive stained nuclei in cortex (**c**) and medulla (**d**). For the quantification of positive Nrf2 nuclei, 10 visual fields of cortical and 3–5 visual fields of medullary kidney sections were analyzed per animal via ImageJ. (**e**) Representative images of double stained kidney sections used for the localization of pNrf2 in kidney cells. Double staining on paraffin-embedded kidney sections was carried out using antibodies against pNrf2 (brown staining) and against calbindin (purple staining), a marker for distal tubule and early collecting duct cells. Examples of pNrf2-positive stained nuclei are indicated by black arrows; white arrows indicate the corresponding section of the tubulus system. Proximal tubules were identified by the presence of the brush border, whereas glomeruli were identified by their capillary tuft (blue circles). Distal tubules and the early collecting duct were visualized by positive calbindin staining. The late collecting duct was identified by the absence of positive calbindin staining and brush border. (**f**–**i**) Quantification of pNrf2-positive nuclei in the four kidney structures related to the number of nuclei in regions positive for the specific kidney cell identifiers in 10 visual fields. For the quantification in the glomerulus, 50 glomeruli were evaluated. Aldo: aldosterone, C: control, Nrf2: nuclear factor erythroid 2-related factor 2, Sulf: sulforaphane, WT: wild type. Data are shown as mean + SEM, *n* = 7–8. * *p* ≤ 0.05 vs. WT-C, ^#^ *p* ≤ 0.05 vs. WT-Sulf, ° *p* ≤ 0.05 vs. WT-Aldo, ^ *p* ≤ 0.05 vs. Nrf2ꜛ-C.

When the phosphorylated form of Nrf2, pNrf2, which is ostensibly regarded as the active form of the transcription factor, is considered, we found its significant increase only in the Aldo-treated Nrf2ꜛ group in the cytosolic fraction (Figure 5a,b). In contrast, in the nuclear and the cytosolic extracts of the Aldo-treated WT mice, interindividual variations prevented the finding of significant changes of pNrf2. Here, immunohistochemical staining revealed a significantly increased amount of pNrf2 only in the cortex of WT-Aldo mice (Figure 5c,d). An even more detailed localization of pNrf2 according to kidney structure or cell type (Figure 5e–i) showed a significant increase in the distal tubule, early and later collecting duct of all Aldo-treated groups and also in the later collecting duct in the Nrf2ꜛ-C group.

**Figure 6:**
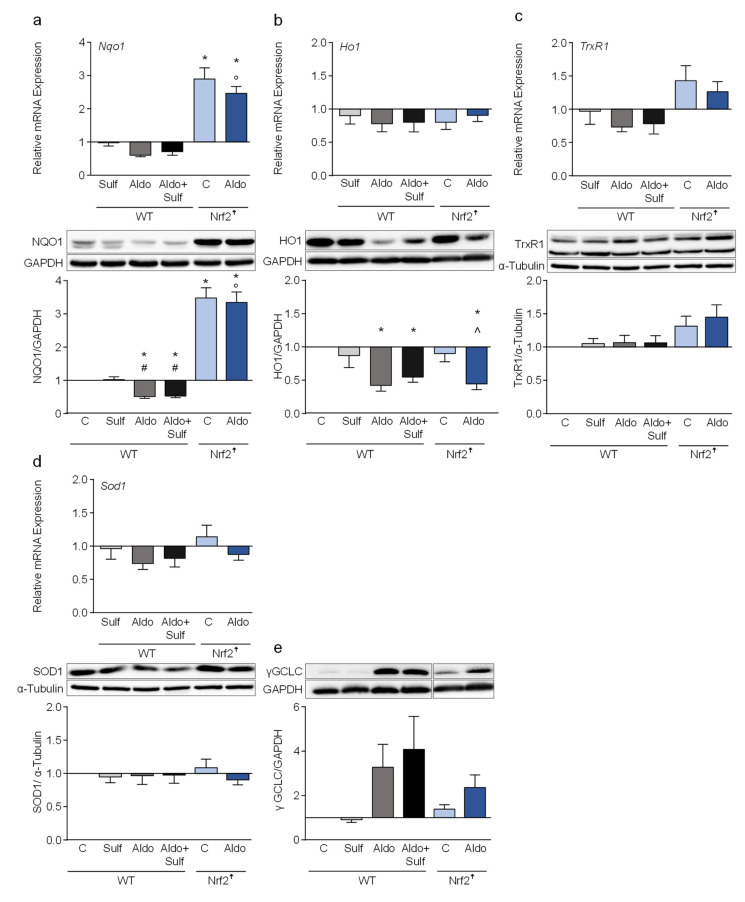
Expression of Nrf2-regulated genes and proteins in Aldo-infused WT and Nrf2ꜛ mice and control mice after 28 days of treatment. Quantification of RT-PCR as well as representative pictures of Western blots and the quantification of band densities measured via ImageJ and related to the housekeepers GAPDH (37 kDa) or α-tubulin (55 kDa) of (**a**) NQO1 (31 kDa), (**b**) HO1 (32 kDa), (**c**) TrxR1 (71 kDa), (**d**) SOD1 (16 kDa) and (**e**) γGCLC (73 kDa) are shown. Aldo: aldosterone, C: control, GAPDH: glyceraldehyde 3-phosphate dehydrogenase, γGCLC: γ-glutamate cysteine ligase catalytic subunit, HO1: heme oxygenase 1, NQO1: NADPH quinone dehydrogenase 1, SOD1: superoxide dismutase 1, Sulf: sulforaphane, TrxR1: thioredoxin reductase 1, WT: wild-type. Data are shown as mean + SEM. *n* = 7–8. * *p* ≤ 0.05 vs. WT-C, ^#^ *p* ≤ 0.05 vs. WT-Sulf, ° *p* ≤ 0.05 vs. WT-Aldo, ^ *p* ≤ 0.05 vs. Nrf2ꜛ-C.

Considering the gene and protein expressions of the target genes of Nrf2, it is striking that only the target exclusively regulated by Nrf2, NADPH quinone dehydrogenase 1 (NQO1), was significantly downregulated at both gene and protein levels in the Aldo-treated WT groups and upregulated in both Nrf2ꜛ groups (Figure 6a). Furthermore, thioredoxin reductase 1 (Trxr1) and superoxide dismutase 1 (Sod1) showed significant downregulation at the gene level in the Aldo-treated WT group and heme oxygenase 1 (HO1) at the protein level in all Aldo-treated groups (Figure 6b–d). The catalytic subunit of glutathione cysteine ligase (γGCLC) showed no difference between groups (Figure 6e).

### 3.6. Keap1-Independent Regulation of Nrf2

**Figure 7:**
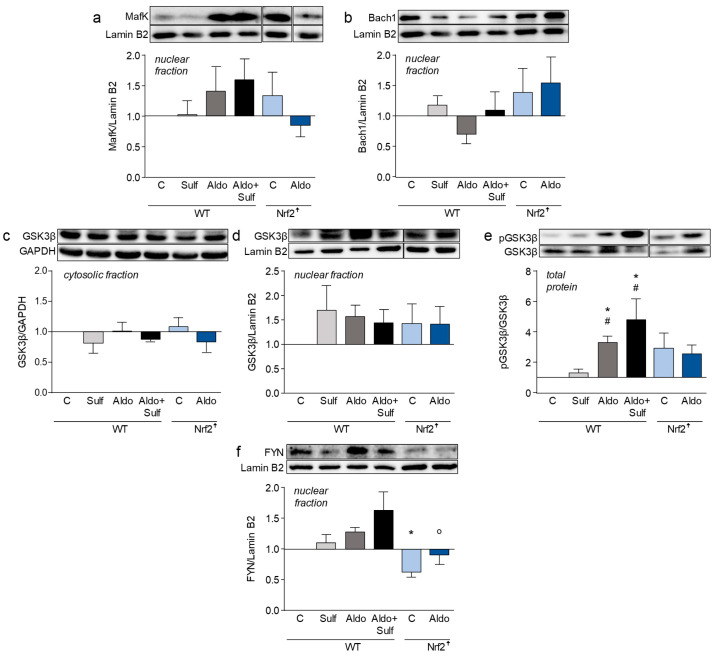
Expression of proteins important for the function of active Nrf2 and of proteins regulating Nrf2 activity in Aldo-infused WT and Nrf2ꜛ mice and control mice after 28 days of treatment. Representative pictures of Western blots and the quantification of band densities measured via ImageJ and related to the nuclear protein lamin B2 (68 kDa) or, in the case of pGSK3β, to non-phosphorylated GSK3β (46 kDa) of (**a**) nuclear localized MafK (18 kDa), (**b**) nuclear localized Bach1 (81 kDa), (**c**) GSK3β (46 kDa) localized in the cytosol, (**d**) nuclear localized GSK3β (46 kDa), (**e**) total phosphorylated GSK3β (46 kDa) and (**f**) nuclear localized FYN (60 kDa) are shown. Aldo: aldosterone, Bach1: BTB and CNC homology 1, basic leucine zipper transcription factor 1, C: control, FYN: Src family tyrosine kinase, GSK3β: glyceraldehyde 3-phosphate dehydrogenase, MafK: musculoaponeurotic fibrosarcoma K, Sulf: sulforaphane, WT: wild-type. Data are shown as mean + SEM, *n* = 7–8. * *p* ≤ 0.05 vs. WT-C, ^#^ *p* ≤ 0.05 vs. WT-Sulf, ° *p* ≤ 0.05 vs. WT-Aldo.

Because downregulation of Nrf2 targets was found despite increased Nrf2 and pNrf2 levels in Aldo-treated WT animals, known negative regulators of Nrf2 activity were examined. The heterodimerization partner of Nrf2, MafK, was not changed in nuclear extracts of Aldo-treated WT animals (Figure 7a). The transcriptional regulator Bach1 is a competitor to Nrf2 in dimerization with MafK, but was also not changed in any group (Figure 7b). The kinase GSK3β, which targets Nrf2 to proteasomal degradation via phosphorylation at Ser 335 and 338, was changed neither in the cytosol (Figure 7c) nor in the nucleus (Figure 7d). In contrast, its inactive form phosphorylated at serine 9 was significantly increased in the cells of both Aldo-treated WT groups (Figure 7e). The Src family tyrosine kinase FYN, which can also mark Nrf2 for proteasomal degradation via phosphorylation, showed a significant reduction in nuclear extracts in both groups of Nrf2 mice compared with WT animals (Figure 7f).

## 4. Discussion

Nrf2 activation was only minimally able to protect kidneys of mice from damage induced by Aldo. Neither pharmacological activation of Nrf2, which proved highly ineffective in this model, nor genetic activation of Nrf2 prevented systemic or local oxidative stress and also had little effect on renal damage markers. In WT animals treated with Aldo, there was broad downregulation of the Nrf2 system.

To examine the effects of Nrf2 activation on moderate renal injury, we chose an Aldo concentration that did not persistently increase blood pressure significantly above normal but produced measurable histological and physiological changes in the kidney [15]. These changes to the kidney were different in the treatment groups and showed higher damage to the glomeruli and moderate protection of the tubules in the Aldo-treated Nrf2ꜛ animals, whereas the Nrf2 activator Sulf appeared to provide mild protection of the glomeruli in the WT mice, but none to their tubuli. Overall, Nrf2ꜛ animals had decreased creatinine clearance, increased albumin excretion and a tendency to hydronephrosis from the outset. Risk factors for the development of hydronephrosis include ureteral stenosis and heart failure and also increased water and sodium intake [31], which was the case for our animals. Hydronephrosis has not been previously reported in the Nrf2ꜛ mouse, but only in a mouse in which Keap1 was specifically deleted in the renal epithelium [32]. Here, all animals studied already suffered from the renal alteration without treatment. In our study, hydronephrosis was more pronounced in the Aldo-treated group, which also had an increased drinking volume. The Nrf2ꜛ mouse seems to be predisposed here, as this phenomenon was not found in the Aldo-treated WT mice despite similar drinking volume, and because a quarter of the Nrf2ꜛ control mice already showed hydronephrosis, possibly caused by the NaCl administered to all mice in the drinking water. All of this suggests a negative effect of Keap1 knockdown on renal development and/or function, which has not been reported previously [27,33,34,35]. Furthermore, we saw increased collagen deposition in the hearts of animals with Nrf2 activation, which may be a contributing factor to the cardiac side effects of the Nrf2 activator CDDO-Me. It is known that complete deletion of Keap1 in Nrf2ꜛ mice leads to death from malnutrition due to hyperkeratosis of the esophagus shortly after birth [36]. Until our report, only studies showing positive effects of Keap1 knockdown in renal damage models, such as protection from tubular damage in reperfusion or ureteral obstruction, had been published [35,37]. Our animals also showed some protection of tubuli, however, only by the genetic modification and not by the activator. This contradicts observations from studies performed with another Nrf2 activator, CDDO-Me, which was able to protect tubules at least during shorter treatment periods [38,39].

The increased Aldo-induced albumin excretion reveals the sensitivity of Nrf2ꜛ animals to glomerular damage, which has already been reported from three other renal injury models and in which increased fibrosis in the glomerulus was observed, as in the animals in this work [34]. There, loss of podocyte processes was additionally seen. Sulf somewhat reduced albuminuria in the WT animals, as previously shown for Sulf and also curcumin in other models [5,11].

As expected for the Nrf2ꜛ mouse strain used, the expression of Keap1 was already decreased by more than 50% by insertion of the loxP sites in the absence of activating Cre expression [33,37]. That the decreased amount of Keap1 basally has no direct effect on Nrf2 expression has also been observed previously by another group, but does not indicate anything about the posttranslational regulation of Nrf2 [37]. Looking closer at the localization of Nrf2, in these groups of animals, as in the preparatory experiment [15], a significantly increased Nrf2 amount was shown in all Aldo-treated groups only in the cortex, not in the medulla of the kidney. Too large interindividual differences among the animals prevented an evaluation of the presence of Nrf2 in the nucleus. Therefore, the presence of phosphorylated and thus usually labeled active pNrf2 was additionally quantified in nuclear and cytosolic extracts but also in the different cells of the kidney. Indeed, phosphorylation is not essential for translocation to the nucleus [40]. Increased phosphorylation of Nrf2 was confirmed in the cortex of Aldo-treated WT animals and, at greater resolution, increased phosphorylation in the nuclei of the distal tubules and collecting duct in all Aldo-treated groups and, at the latter site, also in the Nrf2ꜛ control group. Sulf alone failed to alter phosphorylation status. Differential distribution of Nrf2 but also of other components of the antioxidant defense system within the different nephron segments was reported by several groups, with frequently better equipment found in the proximal tubule [41,42].

Again, as previously reported, Aldo treatment in mice did not induce Nrf2 target genes as we previously observed in rats [11,15], consistent with reports of downregulated Nrf2 targets from the human situation in CKD and other animal CKD models [5,16]. Unexpectedly, based on our previously conducted rat study with Sulf as the Nrf2 activator [11], Sulf had no effect at all on Nrf2 target genes, either alone or in combination with Aldo. It is unlikely that the reason for this was the chosen oral route of administration, which represented less suffering for the animals. Oral application was supposed to compensate for the short half-life of Sulf of about 2 h, which was also the time point where the highest concentration of Sulf was detected in kidneys of mice after a single oral administration [43,44]. This kind of application had worked very well in our rats, in which it was shown that 80% of orally administered Sulf was bioavailable [11,45]. The choice of the Sulf dose used was in the range of published doses which varied from 0.5 to 12.5 mg/kg × day, with one report in which a higher dose of 10 mg/kg × day produced no effects compared with the lower dose of 2 mg/kg × day [46]. As some effects were noted in the animals, it cannot be assumed that the Sulf was not incorporated at all, but possibly the dose was not appropriate for this model. The assumption that Sulf may be less effective in the kidney than CDDO-Me was not expected based on its use and effects in animal models of renal injury [11,47]. That we do not find a clear protective effect against Aldo-induced damage, as seen in other models of renal injury, is partly because of the adverse effects of Aldo on Nrf2 signaling discussed below. On the other hand, the type of renal injury also plays a crucial role. Protective effects of Nrf2 activators have been seen mainly in acute injury and in models that primarily induce tubular injury [35,37,39]. When glomerular damage is involved, reports that find no protection by Nrf2 activation accumulate, as we observe in our animals [34,46]. Thus, it may be that only patients with specific renal diseases that do not involve marked proteinuria benefit from Nrf2 activation.

In contrast to the situation in the WT mice, in the Nrf2ꜛ mice, there was a clear induction of the most specific Nrf2 target, NQO1. Other targets such as HO1 regulated by other transcription factors besides Nrf2 [48] were decreased or only tended to be increased, such as TrxR1 or γGCLC. In most cases, Aldo enhanced the response already seen basally, with the exception of SOD1. Other studies using Nrf2ꜛ mice all showed induction of NQO1 at the protein or mRNA level, suggesting sustained activation of Nrf2 signaling in the kidney [33,34,35,37]. Additionally, microarray analysis showed that gene expression of most Nrf2 target genes was already basally increased in Nrf2ꜛ mice, which included the targets NQO1, TrxR1, γGCLC, and HO1, also studied in this work [35].

Thus, although increased levels of Nrf2 and also of pNrf2 were found in the kidney, no induction of Nrf2 targets was detected in the WT mice. This suggests that either dimerization partners were lacking or not present in the appropriate proportion for the unhindered activity of Nrf2 or pNrf2 in the nucleus, or that the function of Nrf2 was negatively regulated. For example, the formation of a heterodimer with sMaf is essential for the binding of Nrf2 to the ARE [24]. However, sMaf proteins can also form homo- or heterodimers with each other or with other proteins, such as Bach1, which inhibits transcription of ARE-regulated genes [49]. Nguyen et al. demonstrated that overexpression of MafK represses the expression of ARE-dependent catalase in a dose-dependent manner [50]. The MafK level was indeed slightly higher in the nuclear fraction of Aldo-treated WT groups, which may have resulted in an inhibition of expression, whereas no clear picture emerged for Bach1.

The potential negative regulator GSK3β was slightly increased in the nucleus, where its phosphorylation of Nrf2 would lead to its nuclear export with subsequent degradation, whereas no change in GSK3β was detected in the cytosol. In the Aldo-targeted WT groups, even the inactive phosphorylated form of GSK3β, pGSK3β, was significantly increased and therefore cannot be responsible for the decreased expression of Nrf2 targets. Higher expression of GSK3β was observed in a folic acid-induced CKD model in mice after 4 weeks and resulted in increased oxidative stress but lower Nrf2 accumulation in the nucleus and decreased HO1 expression [51]. This study further demonstrated that after initial damage to the kidney, the Nrf2 pathway was activated, but this activation progressively decreased over the next 4 weeks, coupled with an increase in GSK3β protein levels. Furthermore, these effects were also found in renal biopsies from CKD patients [51]. The important role of GSK3β is underlined by a study in which inhibition of GSK3β was able to protect against Aldo-induced inflammation and fibrosis in the kidney and heart of mice [52]. Sulf-only-treated animals showed no significant change in GSK3β or pGSK3β, although Sulf theoretically not only has an effect on the Keap1–Nrf2 interaction but can also affect Nrf2 regulation via deactivation of the GSK3β kinase. Thus, inactivation of GSK3β via its phosphorylation was observed in the heart of Sulf-treated mice [53] and a negative effect of Sulf on GSK3β activity was also shown in diabetic rats [54]. A clearer picture could be obtained if regulators were also examined at the cell type level rather than in total kidney extracts.

The last negative regulator we examined in this work was the kinase FYN, which can eject Nrf2 from the nucleus by phosphorylation and is itself regulated by GSK3β [55]. Here, despite the analysis of whole kidney extracts, there was a significant difference between the WT and Nrf2ꜛ animals in the expression of regulators of Nrf2, because the FYN level was slightly higher in the WT animals, whereas it was already present at a significantly lower level basally in the Nrf2ꜛ-C group and was also significantly decreased compared to the Aldo-WT animals after Aldo treatment. This could explain the difference in Nrf2 target expression between the two mouse strains.

A limitation of our study is that specific Nrf2 activators were not yet available at the time of the experiment. It would be very interesting in the future to investigate the responses of the kidney to the specific protein interaction inhibitors. Furthermore, the very high interindividual variations of the groups were a hindrance to obtaining significant results from more of the parameters studied. The unexpected lack of protection of the kidneys of Aldo-treated animals against damage by Nrf2 activation could be explained with the help of recent publications, the findings of which are supported by our study.

## 5. Conclusions

In conclusion, neither the Nrf2 activator Sulf as used in the model presented here nor genetic upregulation of Nrf2 activity provided protection against Aldo-induced moderate renal injury. In contrast, negative effects of constitutive genetic Nrf2 upregulation were observed with respect to the glomeruli and the filtration barrier, which was also the case, only to a lesser extent, in the Sulf-treated mice. In addition, the hearts of the Sulf-treated animals showed an increase in collagen deposition, as did the animals with genetically activated Nrf2. Treatment with Aldo resulted in extensive inhibition of Nrf2-regulated antioxidant defenses in the kidney. This was not the case with genetic Nrf2 activation for most Nrf2 targets, possibly due to a lower presence of the negative Nrf2 regulator FYN in the nucleus and an inactive GSK3β in the cytosol. Our results highlight the unclear effects of Nrf2 activation on the body in kidney injury. However, we are one step closer to understanding the reduced Nrf2 activity in kidney injury, which may be due to an activated GSK3β/FYN axis.

## Figures and Tables

**Table 1:** Overview of weight, renal function and histological parameters of WT and Nrf2ꜛ mice after 28 days of treatment. Shown in each case is the mean ± SEM. The parameters TSI, MSI and GSI were semi-quantitatively evaluated in the renal cortex of Aldo-treated WT and Nrf2ꜛ mice and their controls. For every parameter, 20 visual fields or 50 glomeruli per animal were analyzed. *n* = 7 for the Nrf2ꜛ-C group for the determination of albumin, KIM-1 and NGAL, *n* = 8 for all other parameters and groups. Aldo: aldosterone, GSI: glomerular sclerosis index, KIM: kidney injury molecule-1, C: control, MSI: mesangiolysis index, NGAL: neutrophil gelatinase-associated lipocalin, Nrf2: nuclear factor erythroid 2-related factor 2, Sulf: sulforaphane, TSI: tubulointerstitial sclerosis index, WT: wild-type. * *p* ≤ 0.05 vs. WT-C, ^#^ *p* ≤ 0.05 vs. WT-Sulf, ° *p* ≤ 0.05 vs. WT-Aldo, ^ *p* ≤ 0.05 vs. Nrf2ꜛ-C.

Parameter	WT-C	WT-Sulf	WT-Aldo	WT-Aldo + Sulf	Nrf2ꜛ-C	Nrf2ꜛ-Aldo
Body weight (g)	29.8 ± 0.5	29.4 ± 0.5	28.3 ± 0.5	29.6 ± 0.9	28.0 ± 0.5	27.5 ± 0.5
Kidney/body weight (‰)	5.8 ± 0.1	5.9 ± 0.1	8.4 ± 0.1 *^#^	8.5 ± 0.2 *^#^	7.6 ± 0.2 *	10.3 ± 0.2 *°^
Heart/body weight (‰)	4.9 ± 0.1	4.8 ± 0.1	5.4 ± 0.2 *^#^	5.3 ± 0.1	5.1 ± 0.2	5.4 ± 0.2 *
Collagen in heart (%)	1.5 ± 0.17	2.5 ± 0.5	1.5 ± 0.3	1.5 ± 0.3	2.0 ± 0.3	3.1 ± 0.7 *°
Hydronephrosis (cases/*n*)	0/8	0/8	0/8	0/8	2/8	4/8
TSI	0.32 ± 0.03	0.33 ± 0.04	0.79 ± 0.04 *^#^	0.73 ± 0.03 *^#^	0.34 ±0.04	0.31 ± 0.03 °
MSI	0.28 ± 0.03	0.34 ± 0.05	0.51 ± 0.05 *	0.35 ± 0.07	0.21 ± 0.04	0.36 ± 0.04 ^
GSI	0.70 ± 0.06	1.14 ± 0.06 *	1.28 ± 0.09 *	1.46 ± 0.14 *	1.19 ± 0.17 *	1.32 ± 0.11 *
Drinking volume (mL/23 h)	6.8 ± 1.3	5.7 ± 0.4	24.2 ± 2.4 *^#^	20.7 ± 1.0 *^#^	7.1 ± 0.8	31.0 ± 4.9 *^
Diuresis (mL/20 h)	3.0 ± 0.6	1.9 ± 0.3	18.6 ± 2.6 *^#^	15.6 ± 1.5 *^#^	2.6 ± 0.7	19.8 ± 5.4 *^
Creatinine in serum (mg/dL)	0.95 ± 0.08	0.96 ± 0.08	1.19 ± 0.45	0.83 ± 0.09	1.26 ± 0.09	1.3 ± 0.15
Creatinine clearance (mL/h)	5.8 ± 0.5	2.9 ± 0.5 *	5.4 ± 1.0	5.6 ± 0.5 ^#^	3.4 ± 0.9	3.5 ± 0.8
Albumin/creatinine (µg/mg)	36 ± 4	43 ± 9	198 ± 41 *^#^	158 ± 25 *^#^	42 ± 8	325 ± 100 *^
KIM-1/creatinine (pg/mg)	409 ± 102	343 ± 72	4203 ± 585 *^#^	4055 ± 643 *^#^	129 ± 39	75 ± 37 °
NGAL/creatinine (ng/mg)	40 ± 3	69 ± 15	272 ± 32 *^#^	257 ± 27 *^#^	51 ± 13	126 ± 32 *°

## Data Availability

The data presented in this study are available in the article and Supplementary Materials.

## Supplementary Materials

The following supporting information can be downloaded at: https://www.mdpi.com/article/10.3390/antiox12030777/s1, Table S1: List of oligonucleotides used for genotyping; Table S2: Primers used for qRT-PCR.

## Abbreviations

8-OHdG	8-hydroxy-2′-deoxyguanosine
γ-H2AX	phosphorylated histone 2AX
Aldo	aldosterone
ARE	antioxidant responsive element
Bach1	BTB and CNC homology 1, basic leucine zipper transcription factor 1
C	control
CKD	chronic kidney disease
EDTA	ethylenediaminetetraacetic acid
FYN	Src family tyrosine kinase
GAPDH	glyceraldehyde 3-phosphate dehydrogenase
γGCLC	γ-glutamate cysteine ligase catalytic subunit
GSK3β	glyceraldehyde 3-phosphate dehydrogenase
pGSK3β	phosphorylated GSK3β
HO1	heme oxygenase 1
Keap1	Kelch-like ECH-associated protein 1
Maf	musculoaponeurotic fibrosarcoma
NADPH	reduced nicotinamide adenine dinucleotide phosphate
NGAL	neutrophil gelatinase-associated lipocalin
Nox2	NADPH oxidase 2 subunit p90
NQO1	NADPH quinone dehydrogenase 1
Nrf2	nuclear factor erythroid 2-related factor 2
pNrf2	phosphorylated nuclear factor erythroid 2-related factor 2
p47phox	NADPH oxidase 2 activator
RAAS	renin–angiotensin–aldosterone system
ROS	reactive oxygen species
SEM	standard error of the mean
SOD1	superoxide dismutase 1
Sulf	sulforaphane
TrxR1	thioredoxin reductase 1
WT	wild type
