# Supplementary material for: Nrf2 Activation Does Not Protect from Aldosterone-Induced Kidney Damage in Mice
Source: Antioxidants (Basel). 2023 Mar 22;12(3):777. doi: 10.3390/antiox12030777 (PMC10044832; doi:10.3390/antiox12030777)
Supplement: Supplementary file 1 [file antioxidants-12-00777-s001.zip › antioxidants-2163955-supplementary.pdf]

antioxidants

Nrf2 activation does not protect from aldosterone-induced kidney damage in mice

Supplemental material

Table S1. List of oligonucleotides used for genotyping.

Gene	Gene ID	Forward (5´-)
Keap1-720s	JN957436	CAGCAGTTAAGGGCACCAATGC
Keap1-925as	JN957436	CCTGCCTCAGCTTCCCATCA
CKOg1	OK586152	CAACGAGAAGCGCGATCACA
Cre1	NC_005856	ACGTTCACCGGCATCAACGT
Cre2	NC_005856	CTGCATTACCGGTCGATGCA

Table S2. Primers used for qRT-PCR.

Gene	Gene ID	Forward (5´-)	Reverse (5´-)
β-actin	NM_007393	GCATTGCTGACAGGATGCAG	CCTGCTTGCTGATCCACATC
Gapdh	NM_008084	TCTCCTGCGACTTCAACA	TCTCTTGCTCAGTGTCCTT
Gclm	NM_008129	TTCTCGGGTGAGGTTTCTGC	AACGAGGGAGCTGTTTCCTG
Gpx1	NM_008160	TTGGTGATTACTGGCTGC	TGATATTCAGCACTTTATTCTTAGTAG
Ho-1	NM_010442	CCAGAGTCCCTCACAGAT	CCCAAGAGAAGAGAGCCA
Keap1	NM_016679	ACGACGTGGAGACAGAGACC	ATCAATTTGCTTCCGACAGG
Nox2	NM_007807	GCGGTGTGCAGTGCTATCAT	GGTTCCAGTGCGTGTTGCT
Nqo1	NM_008706	GGCCGATTCAGAGTGGCAT	CCAGACGGTTTCCAGACGTT
Nrf2	NM_010902	GCAACTCCAGAAGGAACAGG	AGGCATCTTGTTTGGCAATG
Sod1	NM_011434	ACCAGTTGTGTTGTCAGG	TTTCTTAGAGTGAGGATTAAAATGAG
TrxR1	NM_015762	CAGTTCGTCCCAACGAAAAT	GCACATTGGTCTGCTCTTCA

Copyright: © 2022 by the authors. Submitted for possible open access publication under the terms and conditions of the Creative Commons Attribution (CC BY) license (https://creativecommons.org/licenses/by/4.0/).
